# Influence of Undergarments on the Comfort Level of Scoliosis Brace Wearers

**DOI:** 10.3390/ma16175925

**Published:** 2023-08-30

**Authors:** Orsolya Nagy Szabó, Jelka Geršak, András Koleszár, Marianna Halász

**Affiliations:** 1 Sándor Rejtő Faculty of Light Industry and Environmental Protection Engineering, Institute for Industrial Product Design, Óbuda University, Doberdó út 6., H-1034 Budapest, Hungary; szabo.orsolya@uni-obuda.hu (O.N.S.); koleszar.andras@uni-obuda.hu (A.K.); 2 Faculty of Mechanical Engineering, Research and Innovation Centre for Design and Clothing Science, University of Maribor, Smetanova ulica 17, SI-2000 Maribor, Slovenia; jelka.gersak@um.si

**Keywords:** scoliosis brace, clothing physiology, undergarment, knitted fabrics, textile material testing, climate chamber

## Abstract

Bracing has proven to be an effective method for the conventional treatment of scoliosis in young people. A brace, a therapeutic device, covers the upper body and promotes healing by applying pressure to specific areas. However, wearing a scoliosis brace negatively affects the user’s thermo-physiological well-being and often leads to discomfort. In this study, we investigated the influence of T-shirts as an undergarment on the thermo-physiological well-being of the brace wearer. For this purpose, we performed a comparative analysis of six T-shirts made from different special knitted fabrics. We carried out wearing tests in a computer-controlled climate chamber according to a predetermined protocol. The test subject wore the orthopedic brace over the different T-shirts at three different temperatures. The results indicate that the knitted fabrics of undergarments and environmental conditions considerably impact the wearer’s thermo-physiological comfort. In the tests, the T-shirts made from the selected functional fabrics performed very well. The T-shirt made from the classic cotton fabric containing elastane yarn also performed well and was the most environmentally friendly. Currently, due to its lower price and easier availability, this cotton T-shirt can be recommended for wearing under a scoliosis brace.

## 1. Introduction

The number of people with scoliosis is increasing year by year [1]. Scoliosis is regarded as a three-dimensional structural deformity of the spine and trunk, which may worsen during growth [1,2,3]. Scoliosis is most prominent in adolescence (10–16 years) and is more common among girls. Improvement can be achieved with high corrective braces [4,5,6,7]. The brace applies external corrective forces to the special points of the trunk to halt the progression of the abnormal spinal curvature, correct it during growth, or to avoid further progression of an already established pathological curve in adulthood [8].

Various braces are available with different approaches and outcomes [4]. Biomechanically, correction may vary according to brace type. Each brace is tolerated differently, which may affect compliance [9,10]. The brace is manufactured according to the patient’s measurements and disease type by an orthotist. When making a brace, the orthotist chooses the pressure points of the brace for the healing effect so that when a patient wants to avoid the discomfort they cause, they have to use their own muscle power to bring and hold their body precisely in the correct position. If the patient wears the brace for a sufficient time, usually several years, improvement and even complete recovery are possible [11,12,13,14].

The braces usually are made from a special thermoplastic polymer (high- and/or low-density polyethylene, copolymer, or modified polyethylene) and fit around the upper body. They are worn over undergarments every day. Some are worn overnight; some are worn 23 h a day [15,16,17].

Although braces for scoliosis today are more comfortable than ever before, they still have a low compliance rate for various physical, physiological, and emotional reasons [18]. Patients who wear orthopedic braces are also faced with specific problems, such as heat, sweat, heaviness, stiffness, and skin discomfort or problems. Therefore, this research investigates the influence of underwear and activity dynamics on thermo-physiological comfort when the orthopedic braces are worn in a warm environment, which corresponds to high summer temperatures between 25 °C and 32 °C.

Several studies have been conducted on creating fashionable undergarments for brace wearers [19,20,21]. Their functional characteristics can be achieved by designing the cutting lines to avoid the pressure points of the brace and using unique patterns created with body data of 3D optical scanning [22,23,24]. Still, the design alone is not enough to reduce physiological inconvenience.

Much research has been conducted in the field of thermo-physiology addressing the thermal comfort level of special devices and clothing [25,26,27], primarily thermal protective clothing, most often firefighter clothing [28,29,30]. The thermo-physiological comfort of firefighters, workers in some industries, soldiers, or racing car drivers and their safety is an extremely important topic. The problems associated with clothing physiology of young people forced to wear braces for spine treatment is similar, but we did not find any research on this topic.

Much research is devoted to studying functional materials [31], especially those containing microencapsulated phase-change materials (PCMs). Good results have been reported with these materials in thermal protective clothing, even though the PCM capsules have a cooling or heating effect of only approx. 15 min long [26,28,29,30,32,33]. In addition to materials containing PCM capsules, functional textiles developed specifically for better moisture wicking are also extensively researched, for example, artificial fibers that have a specially shaped cross-section [29,34,35].

Our research aims to reduce the inconvenience of wearing a brace while retaining its essential functions. We set out to improve patients’ physiological and mental well-being by creating fashionable undergarments with functional textiles.

A brace, made from a polymeric material, is a thick, continuous, non-porous layer around the upper body; wearing a brace hinders sweat evaporation and heat dissipation, so the user may experience an unpleasant microclimate during physical exertion or when in a warm environment. Therefore, major requirements for the undergarment are its moister absorption attributes, air permeability, and temperature balancing capability.

The undergarment worn under a brace must fit one’s body to avoid it from wrinkling or riding up. Wrinkling and riding up are uncomfortable even in normal circumstances, but if they occur under a brace, they can cause pain or produce bruises under the pressure points of the brace. Therefore, it is of primary importance to use elastic materials and create a unique design to make the undergarment act as a ‘second skin’ without wrinkling, running, or bunching up under the brace. These inconveniences can be avoided with the use of a highly elastic fabric that can ensure that the undergarment fits the body perfectly and can change its shape to adapt to the body and its movements.

Our investigation focused on the selection of fabrics for undergarments. We studied whether the comfort level of wearing undershirts under an orthopedic brace could be affected by the kinds and properties of the fabric of the undergarment. Our aim was to find a fabric with the best thermo-physiological comfort and, if possible, the most environmentally friendly fabric for the undershirts worn under an orthopedic brace [36].

## 2. Materials

We based the experiments on a special construction of T-shirts as the first layer to be worn under a scoliosis brace in a hot environment. The textiles of T-shirts are different knitted fabrics. The most important criteria for selecting the textiles were appropriate thermo-physiological properties and highly elastic behavior. Based on these criteria, we selected six textiles, one classic cotton knitted fabric containing elastane yarn (Cotton) and five functional knitted fabrics. For functional knitted fabrics, we chose a high-performance moisture-wicking fabric (MW-C01) and four different types of fabrics containing PCM microcapsules (OU-W02, OU-F03, OU-B04, and OU-C05). We bought the Cotton and the OU-C05 fabrics in a store, and the other four from the manufacturer for our research purpose. We assumed that these fabrics would be suitable for wearing under a brace. Table 1 shows the basic properties of these knitted fabrics.

We examined the most important physical characteristics of the selected materials in terms of comfort level: water absorption, speed of water-wicking, thickness loss due to abrasion, air permeability, thermal conductivity, and deformability. Table 2 contains these physical characteristics for each fabric, which we determined as the average of 5 tests in each case. The data were measured specifically for the comparison of the tested materials.

Water absorption ability was measured according to the AATCC TM195-2012 [37] standard. During the test, the sample was immersed in water for 20 min and then sandwiched between two sheets of blotting paper to remove excess moisture. We measured the dry and wet weight of samples, and expressed water absorption as a percentage increase in mass (Table 2).

Water-wicking speed was evaluated according to the AATCC TM 197-2013 [38] standard. Wicking refers to spreading water through a given area via capillary action in a material. During the test, the samples were hung vertically, and 10 mm of them were immersed in water. After immersion, the wet area—wicking height—was measured from the water level every 5 min. The results of wicking speed are presented in Table 2.

The thickness loss of knitted fabrics due to abrasion was measured according to the ASTM D4158 [39] standard using the TKI Abrasion Tester. Considering the purpose of use, we chose abrasion with the brace material and the crossing direction movement, which is the most demanding among the movement combinations. Recourse speed was 60 cycles per minute for 30 min with a friction force of 10 N. The results of thickness loss are shown in Table 2.

We measured air permeability according to the ISO 9237:1999 [40] standard using the Metefem FF-12 7236-038 device at a pressure difference of 100 Pa. The results of air permeability tests are presented in Table 2.

We performed the thermal conductivity tests using the KES-F7 Thermo Labo II device for thermal properties (Table 2).

Fabric elasticity is an important parameter, which plays a key role, particularly in the case of tight-fitting garments. Tight-fitting garments of elastic fabric are tailored to a smaller size than the actual size of the wearer’s body. Size reduction is usually 5 percent in body length and 15 percent in circumference, but experience has shown that this amount may not be suitable for some fabrics. To specify these values for the selected fabrics, we also performed tensile tests (with a Textenzer tensile tester device) using the Grab testing method on partially gripped specimens according to the ISO 13934-2:2000 [41] standard. Each specimen was 100 × 150 mm with a gauge length of 100 mm. Based on practical experience, we determined the specific tensile force in both directions where the T-shirt fits the body but is still comfortable for the wearer. These values are 0.5 N/50 mm in the wale (in body length) direction and 2.8 N/50 mm in the course (in circumference) direction. Specific elongation values measured at these specific tensile force values are shown in Table 2.

**Figure 1:**
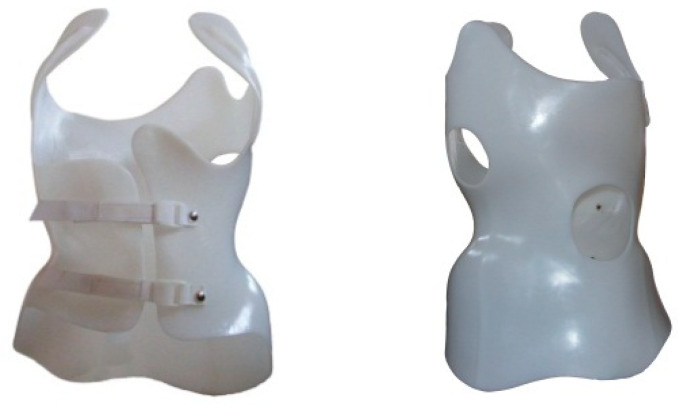
A Chêneau brace, front and back.

The Chêneau brace we used is made of a 5 mm thick HDPE (Mass: 0.941 g/cm^3^) polymer plate. The brace weighs 0.9 to 1.5 kg depending on size. The HDPE polymer plate lacks vapor permeability or moisture-wicking characteristics and is essentially a thermal insulation material. Figure 1 shows a Chêneau brace.

## 3. Methods

These tests aimed to assess the thermo-physiological properties of the undergarment made from selected knitted fabrics, when worn under a brace.

**Figure 2:**
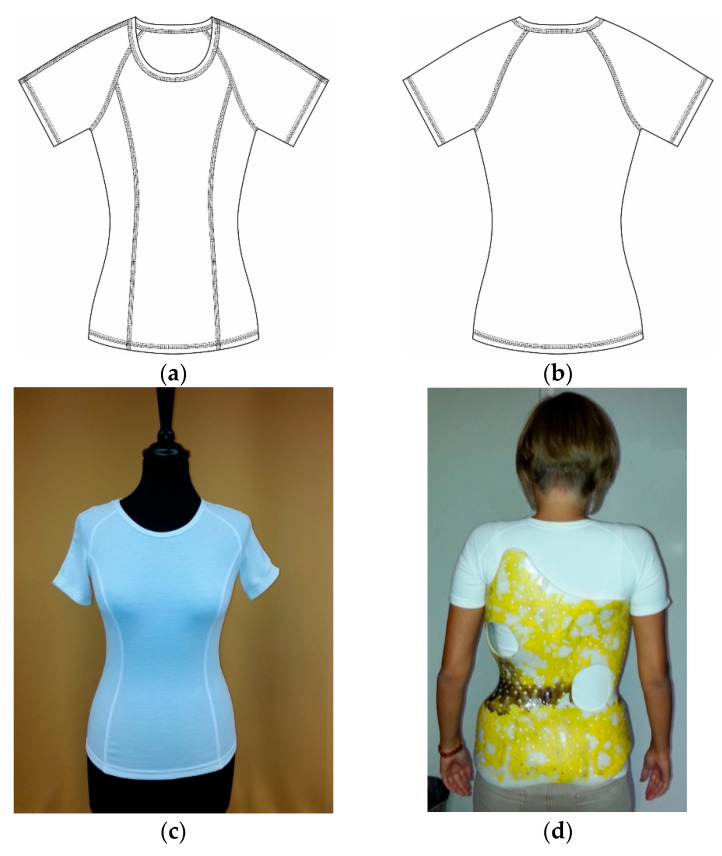
The T-shirt prototype. Front view (**a**), back view (**b**), front view on the mannequin (**c**), back view on a girl with a decorated brace (**d**).

For this purpose, we created a T-shirt prototype pattern (Figure 2).

The special feature of the prototype is that its seam lines avoid the pressure points of the brace, which improves the wearing comfort of the T-shirt.

We could not use brace wearers as test persons due to their young age. Therefore, during the tests, one member of our research team assumed the role of the test person, and she performed the tests in the climate chamber on herself. Salus Orthopaedics Technology Ltd. agreed to make a personalized Chêneau brace for her [15]. Since brace is an expensive orthopedic device, we were only able to use this one test subject. Based on the designed T-shirt prototype, we made a T-shirt from each selected knitted fabric for the test person in her size.

**Figure 3:**
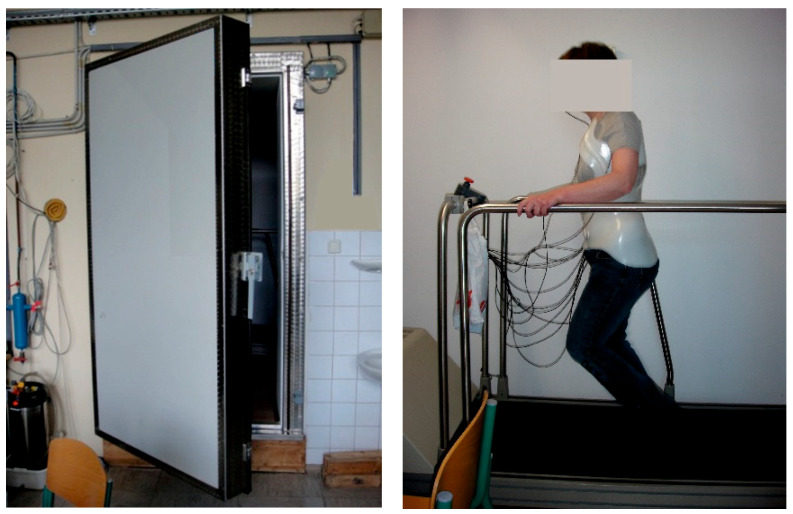
The climate chamber with the treadmill and the test person wearing the brace.

In this study, we carried out wearing tests during which the test person wore the brace over the T-shirts we made. According to a predetermined protocol, we completed the tests in a computer-controlled climate chamber, which provides artificially created ambient conditions, that is, a constant temperature, relative humidity and air velocity. The climate chamber contains a treadmill, a small table, a chair, an alarm and a video camera that transmits the image of the test subject for continually observation (Figure 3).

During the tests in the climate chamber, there were three different ambient temperatures (25 °C, 28 °C, and 32 °C), a constant relative humidity of 50%, and an air velocity of 0.5 m/s. At each of the three ambient temperatures, we performed six tests, during which the test subject wore one of the six T-shirts under the brace; therefore, we performed a total of 18 tests.

In this study, we attempted to simulate the activity of a person who wears a brace. In each activity, we gave sufficient time for the test person to adapt to the thermal environment. The total duration of the test was determined based on the preliminary trials and the mental/physical comfort of the brace wearer.

During testing, the test subject wore T-shirts made from the selected knitted fabrics under her personalized Chêneau brace. Each test lasted 75 min based on the pre-determined protocol, which included the following:

- 15 min of preparation and acclimatization without data collection;
- 20 min of rest (seated position);
- 20 min of walking at a speed of 2.5 km/h on the treadmill;
- 15 min of rest (seated position);
- 5 min of walking on the treadmill at a speed of 3.5 km/h.

**Figure 4:**
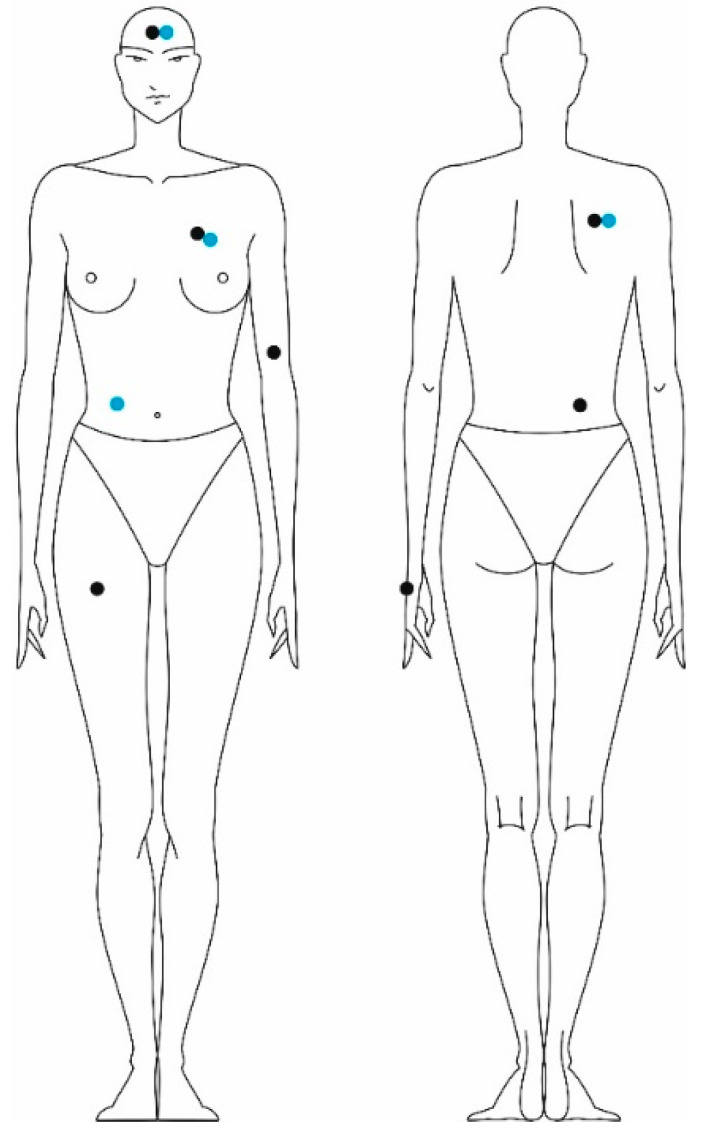
The locations where the skin temperature and relative humidity were measured.

We examined the thermo-physiological parameters using the MSR (Modular Signal Recorder) measuring device from MSR Electronics GmbH, Seuzach, Switzerland. The MSR device is a modular unit for measuring various physiological parameters, such as skin temperature and humidity at the surface of the skin (microclimate humidity). We measured skin temperature at seven different locations on the body and the humidity on the skin surface at four different locations on the body according to EN ISO 9886: 2004 [42] (Figure 4).

To estimate the sweat rate, the test subject weighed her own mass naked before and after testing. The subject’s clothing was also weighed before and after testing. Based on the subject’s weight loss and the clothing ensemble’s weight gain (through absorption of the subject’s sweat during exercise), we determined the loss of the test person’s body fluid due to sweating.

## 4. Results

The results show that the undergarment made from the selected knitted fabrics, and environmental conditions considerably impact the wearer’s thermo-physiological comfort.

**Figure 5:**
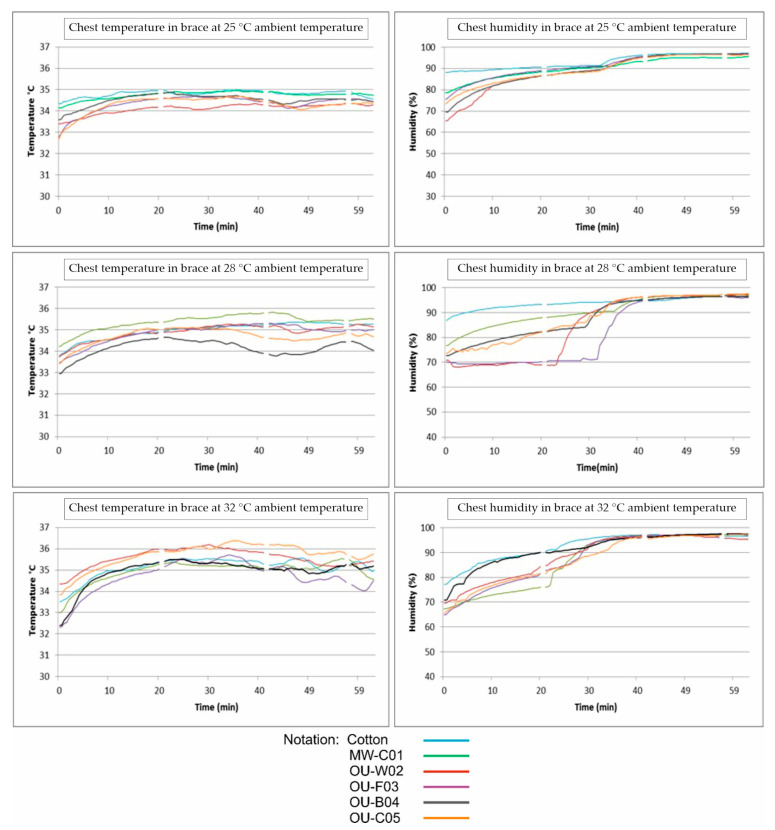
Skin temperature and relative humidity at the skin surface of the test subject as a function of time, at the chest measurement location, at three different ambient temperatures.

Skin temperature and relative humidity at the skin surface were also measured in the measuring point on the chest. Figure 5 shows the results in this measuring point at three ambient temperatures.

The analysis of skin temperature shows that with higher ambient temperature, skin temperature increases. The results also show that the undergarment made from Cotton knitted fabric provided similar results to the undergarments made from fabrics containing phase-changing materials. At the beginning of the test, the undergarment with built-in PCM microcapsules slows down the increase in temperature. However, after the PCM melts, it shows no effect on body temperature. At the beginning, the humidity values show some difference, but these minor differences become insignificant after a short time.

In the course of the test, we obtained well-evaluable results by measuring the weight of the test person’s body and clothing.

Most heat is dissipated through the skin. The brace prevents this on a large part of the upper body because it prevents the evaporation of sweat, which would cool the body. Therefore, the undergarment must absorb the sweat released by the body which cannot evaporate. We measured the decrease in the body weight of the test person during the time spent in the climate chamber and the increase in weight of the T-shirt after soaking up moisture.

**Figure 6:**
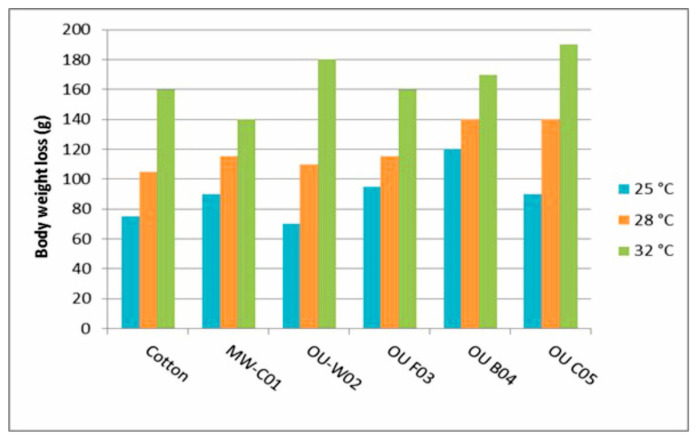
The effect of different T-shirt fabrics on body weight loss at different temperatures.

**Figure 7:**
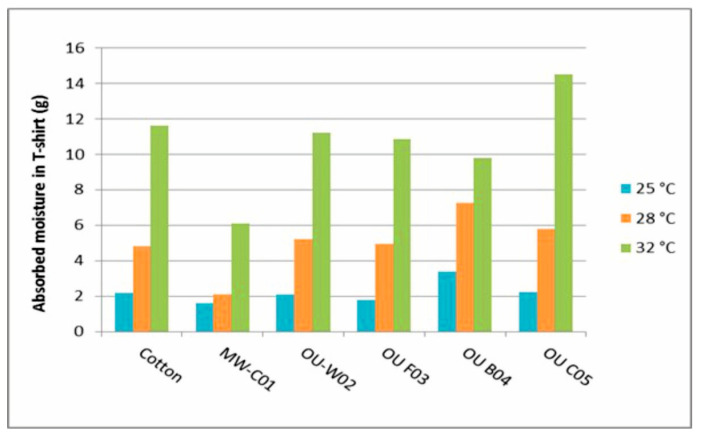
The effect of different T-shirt fabrics on the amount of moisture absorbed at different temperatures.

Less moisture picked up by a T-shirt and a lower body weight loss of the test person during a test means that the test person sweated less, so this T-shirt provides a higher level of comfort. The decrease in body weight is shown in Figure 6, and the weight gain of T-shirts due to moisture absorption is shown in Figure 7.

The measurement results show that the body weight decrease depends on the properties of the fabric of the T-shirt. Weight reduction was between 70 and 120 g at the ambient temperature of 25 °C, between 105 and 140 g at 28 °C, and between 160 and 190 g at 32 °C, depending on the fabric of the T-shirt worn. The body weight of the test person mostly decreased the least at all ambient temperatures when she wore the Cotton T-shirt.

Figure 7 shows that the amount of moisture absorbed by the T-shirts depends also on the properties of the knitted fabric. Moisture absorption varied between 1.5 and 3.7 g at the ambient temperature of 25 °C, between 2.0 and 7.5 g at 28 °C, and between 6.0 and 14.5 g at 32 °C, depending on the T-shirt worn. The T-shirt made from high-performance moisture-wicking fabric (MW-CO1) had the lowest amount of absorbed moisture. The T-shirt made from the OU-C05 fabric at 32 °C and the T-shirt made from the OU-B04 fabric at 25 °C and at 28 °C had the highest amount of absorbed moisture.

## 5. Subjective Assessment

We made T-shirts from the aforementioned selected knitted fabrics, which were worn by 12 people. The test subjects in this examination were girls of age 16 to 18, all wearing braces. The girls voluntarily and willingly agreed to wear the T-shirts for the test. The girls wore the T-shirts under ordinary conditions in a special four-day long camp organized for the brace wearers. According to the prototype pattern, we made four T-shirts from the selected fabrics for the four days, for every girl specific to their size.

Since only 4 days were available for this wearing test, two fabrics had to be omitted from the test. The T-shirt made from the MW-C01 material was very uncomfortable during the climate chamber tests, so we did not make T-shirts from this material for the girls. The other fabric we omitted was the OU-W02 material because its composition and behavior are similar to those of the OU-B04 material. Therefore, we would presumably get similar results during the wearing test, as in the case of the OU-B04 material.

The girls wore each T-shirt for one day, so the wearing conditions were the same. They rated the T-shirts on the last day of the camp. The testers’ feedback was positive; the girls were happy that these T-shirts suited their special needs more than the ones available off the shelf. Some of them have been wearing these T-shirts since then. The girls provided feedback on their experience by filling out a questionnaire. In the questionnaire, we asked the girls about the quality of the material: the feel of the material, the fit to the body, and the comfort of wearing. Table 3, Table 4 and Table 5 contain their answers to the relevant questions of the questionnaire.

The girls thought that in wearing comfort and touch of fabric, the OU-B04 material was the best, and the OU-C05 material was the least good. The OU-B04 material is a soft and elastic fabric, which is comfortable to wear, according to the testers. In comfort and touch, Cotton was the second best after OU-B04.

One of the most important criteria of a T-shirt is to fit the body perfectly. In designing undergarments for brace wearers, it is of primary importance that the final product does not wrinkle or run up under the brace and fits the body well.

T-shirts manufactured from Cotton knitted fabrics are close-fitting and follow the body without wrinkles. The T-shirt made from the OU-C05 knitted fabric proved to be too loose, and as a result, wrinkles appeared. Therefore, it did not meet the requirements. With a bigger under-tailoring value, it is possible to improve the fitting of the OU-F03 and OU-B04 fabrics.

During the test wearing, the brace did not damage the T-shirts.

To summarize, the girls preferred the elasticity and the thermo-physiological comfort of the Cotton T-shirt and the thermo-physiological comfort and soft touch of the OU-B04 T-shirt. According to the girls, the OU-F03 knitted fabric proved to be mediocre in every respect, and the OU-C05 knitted fabric was the worst.

## 6. Discussion

We chose the six tested fabrics because we believed that they might meet the special requirements of undergarment fabrics worn under a brace.

The evaluation of the physical characteristics of the selected fabrics, the tests conducted in the climate chamber, and the subjective assessment of the girls wearing braces provide a complete and detailed picture of the examined fabrics.

Therefore, it is not surprising that the fabrics (except OU-C05) performed well in the tests. The results confirmed our opinion that Cotton, an environmentally friendly, natural, and conventional fabric, can compete with the recently developed functional fabrics.

The significant aspects of the evaluation were the following:

- General thermo-physiological aspects: Good moisture absorption and moisture transmission properties, high air permeability, and advantageous temperature balancing capability.
- Mechanical properties: High elastic elongation and high abrasion resistance.
- Thermo-physiological test results: Lower body weight decrease, and lower perspiration rate of the test person, and suitability of the T-shirts at different temperatures.
- Subjective evaluation by the test persons.
- Availability off the shelf.
- Acceptable price-to-value ratio.
- Being environmentally friendly.

We ranked the fabrics according to different priorities.

Although the MW-C01 fabric was the best regarding moisture absorption and air permeability, and performed well in the climate chamber test as well, it is the worst regarding elastic deformation because it does not contain elastane fibers.

Although the Cotton fabric performed worst in moisture absorption and air permeability, it was good in elastic deformation and was balanced in the climate chamber test.

Among the fabrics containing PCMs, the OU-B04 fabric was the best, while the OU-C05 fabric was the weakest.

Another aspect was the opinions of the girls who performed the wear test. They liked the Cotton and OU-B04 T-shirts best. Due to the small elastic deformation, the MW-C01 fabric probably would not have won the girls’ favor.

The last three aspects tip the balance toward the Cotton fabric. This fabric is the cheapest, it is easily available, and it is clearly the most environmentally friendly of the examined fabrics [43]. Since this fabric had a balanced performance in the tests, currently, the Cotton T-shirt can be recommended for wearing under a brace.

The results of our study are of limited validity as the climate chamber tests were performed with the participation of only one test person, and each T-shirt was tested only once at each temperature. Therefore, we have no data for statistical evaluation. Nevertheless, we believe our results are valuable, provide good data for orientation, and they are a good starting point for further more comprehensive investigations.

## 7. Conclusions

In this study, we researched how undergarment fabrics can influence a brace wearer’s thermo-physiological comfort level. We compared six T-shirts made from different special knitted fabrics. In the wearing tests, the test person wore the brace over the undergarment. The tests were performed in a computer-controlled climate chamber at three different temperatures. In addition, we also received subjective assessments from teenage girls wearing braces, who wore our shirts under their braces for a few days. The results indicate that the knitted fabrics of the undergarments and environmental conditions considerably impact the wearer’s thermo-physiological comfort. The recently developed functional, high-performance moisture-wicking fabric and the functional fabrics containing PCMs performed very well in most aspects; therefore, they are suitable for wearing under a brace. However, unfortunately, they are expensive and currently hard to acquire. The T-shirt made from the classic Cotton fabric containing elastane yarn also performed well in the tests. Since this fabric is currently the most environmentally friendly, the cheapest, and the most easily available of all the examined fabrics, it can be recommended for wearing under a brace.

## Figures and Tables

**Table 1:** Basic properties of the knitted fabrics.

Kind of Knitted Fabric	Textile Composition	Structure of Fabrics	Thick-ness [mm]	Mass [g/m^2^]	Yarn Count [Tex]	Course Density [Piece/10 mm]	Wale Density [Piece/10 mm]	PCM Melt Peak [°C] (Nominal Data)	PCM Storage Capacity [J/g] (Nominal Data)
Cotton	97% cotton 3% elastane	Single weft-knitted	0.64	242	19.3	26	16	-	-
MW-C01	100% PES with silver ions, and special filament cross-section	Double pique-knitted	0.73	168	13.0	17	12	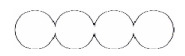 Special filament cross-section	
OU-W02	66% cotton 28% viscose with PCM capsules 6% elastane	Single weft-knitted	0.64	198	15.7	28	18	24–27	>2.5
OU-F03	95% cotton 5% elastane coating: silicon with PCM capsules	Single weft-knitted	0.65	146	16.4	22	17	26–30	>6
OU-B04	66% lyocell 28% viscose with PCM capsules 6% elastane	Single weft-knitted	0.66	175	15.6	26	17	24–27	>2.5
OU-C05	59% PES 39% viscose with PCM capsules 2% elastane	Rib weft-knitted	0.68	192	22.4	20	16	no data	no data

**Table 2:** The physical characteristics of selected knitted fabrics.

Kind of Knitted Fabric	Water Absorption m_w_ [%]	Water- Wicking Speed v_w_ [mm/min]	Thickness Loss h_av_ [%]	Air Perme-ability Q $\left[\frac{{dm}^{3}}{m^{2}\cdot s}\right]$	Thermal Conductivity $\lambda \;\left[\frac{W}{mK}\right]$	Deformation Due to Tensile Force/50 mm	
						0.5 N Wale Direction ε [%]	2.8 N Course Direction ε [%]
Cotton	186	3.6	7.8	14.8	0.0565	5.2	17.7
MW-C01	300	3.5	4.1	210.3	0.0508	2.4	11.6
OU-W02	233	3.8	6.3	28.9	0.0513	5.7	22.3
OU-F03	254	2.7	12.7	51.7	0.0515	8.6	26.4
OU-B04	218	1.3	8.9	99.6	0.0525	9.1	27.6
OU-C05	226	2.6	4.4	141.4	0.0516	3.3	27.5

**Table 3:** Ranking of the T-shirts according to the touch of the fabric. (1 is the best and 4 is the worst).

Kind of Knitted Fabric	Order According to the Girls	Sum	Ranking
Cotton	1, 1, 2, 2, 2, 2, 2, 3, 3, 3, 3, 3	27	2
OU-F03	1, 1, 2, 2, 2, 3, 3, 3, 3, 3, 3, 3	29	3
OU-B04	1, 1, 1, 1, 1, 1, 1, 1, 2, 2, 2, 2	16	1
OU-C05	4, 4, 4, 4, 4, 4, 4, 4, 4, 4, 4, 4	48	4

**Table 4:** Ranking of the T-shirts according to the comfort of wearing them. (1 is the best and 4 is the worst).

Kind of Knitted Fabric	Order According to the Girls	Sum	Ranking
Cotton	1, 1, 1, 1, 2, 2, 2, 2, 3, 3, 3, 3	24	2
OU-F03	2, 2, 2, 2, 3, 3, 3, 3, 3, 3, 3, 3	32	3
OU-B04	1, 1, 1, 1, 1, 1, 1, 1, 2, 2, 2, 2	16	1
OU-C05	4, 4, 4, 4, 4, 4, 4, 4, 4, 4, 4, 4	48	4

**Table 5:** Ranking of the T-shirts according to fit. (1 is the best and 4 is the worst).

Kind of Knitted Fabric	Order According to the Girls	Sum	Ranking
Cotton	1, 1, 1, 1, 1, 1, 1, 2, 2, 2, 3, 3	19	1
OU-F03	2, 2, 2, 2, 2, 2, 2, 3, 3, 3, 3, 3	29	3
OU-B04	1, 1, 1, 1, 1, 2, 2, 3, 3, 3, 3, 3	24	2
OU-C05	4, 4, 4, 4, 4, 4, 4, 4, 4, 4, 4, 4	48	4

## Data Availability

Detailed data can be provided by the corresponding author upon request.
